# Hormesis: a fundamental concept in biology

**DOI:** 10.15698/mic2014.05.145

**Published:** 2014-04-23

**Authors:** Edward J. Calabrese

**Affiliations:** 1 Department of Public Health, Environmental Health Sciences, Morrill I, N344; Amherst, MA 01003 USA.

## Abstract

This paper assesses the hormesis dose response concept, including its historical foundations, frequency, generality, quantitative features, mechanistic basis and biomedical, pharmaceutical and environmental health implications. The hormetic dose response is highly generalizable, being independent of biology model (i.e. common from plants to humans), level of biological organization (i.e. cell, organ and organism), endpoint, inducing agent and mechanism, providing the first general and quantitative description of plasticity. The hormetic dose response describes the limits to which integrative endpoints (e.g. cell proliferation, cell migration, growth patterns, tissue repair, aging processes, complex behaviors such as anxiety, learning, memory, and stress, preconditioning responses, and numerous adaptive responses) can be modulated (i.e., enhanced or diminished) by pharmaceutical, chemical and physical means. Thus, the hormesis concept is a fundamental concept in biology with a wide range of biological implications and biomedical applications.

## INTRODUCTION

Hormesis is a biphasic dose response phenomenon, characterized by a low dose stimulation and a high dose inhibition. The term hormesis first entered the scientific lexicon in 1943 by Chester Southam and John Ehrlich 1 based on their observations that extracts from the Red Cedar tree enhanced the metabolism of fungal species at low concentrations. While this was the first use of the term hormesis, from the Greek to excite, there was a substantial history of biphasic dose responses from the 1880s onward with the first experimental evidence concerning the effects of numerous disinfectants on the metabolism of yeast 2,3. These findings were confirmed and extended by others with most research using plant or microbiological models. Such information became incorporated into various textbooks in botany 4,5 and microbiology 6,7,8,9 during the early to mid portions of the 20^th^ century [See 10,11,12,13,14 for a detailed summarization]. Considerable efforts in the area of plant biology and agriculture assessed how this biphasic dose response concept could enhance crop production with exposures in the low dose range.

## HORMESIS: HISTORICAL CONFLICTS

Despite considerable research documenting the occurrence of biphasic dose responses during these early years, this concept became embroiled in the long dispute between homeopathy and what we call today traditional medicine. Soon after Schulz first observed the occurrence of the biphasic dose response with yeast, he came to believe that he had discovered the explanatory principle of homeopathy 15. Since Schulz was a traditionally trained physician and professor of pharmacology he came under immediate and intense criticism by his medical colleagues at the University of Greifswald and others in the traditional medicine community and soon became marginalized by his peers. However, what was significant was that Schulz essentially handed homeopathy the biphasic dose response, thereby scooping traditional medicine on the concept of the dose response. In retrospect it is not difficult to understand why traditional medicine and its core disciplines, such as pharmacology, did not accept Schulz’s biphasic dose response. It was not possible for traditional medicine to accept the dose response of their intense rival. Doing so would provide credibility to homeopathy. In fact, traditional medicine organized to discredit Schulz and his biphasic dose response model all the while attacking homeopathy, which, at the time, was fairly formidable 16,17,18,19. Eventually traditional medicine would adopt its own dose response model, gravitating to the threshold dose response as a centerpiece in pharmacology, therapeutics and toxicology.

The 20^th^ century would see a rapid reversal in the fortunes of homeopathy, becoming highly marginalized, whereas traditional medicine would become enormously successful 15. In 1900 there were two dozen homeopathic medical schools in the United States. Some 25 years later there were but three. Mirroring these institutional changes, the biphasic dose response became marginalized, omitted from textbooks, curricula, research funding and regulatory procedures. In essence, it received the equivalent of a scientific and academic death sentence. By the time of Schulz's death in 1932 his biphasic dose response model had little standing in the medical community, some five decades after his initial discovery. In contrast, the threshold dose response became broadly accepted, incorporated into all pharmacology and toxicology textbooks and became the basic dose response model for government mandated hazard assessments for chemicals, drugs and radiation.

Yet the concept of the biphasic dose response did not die. As noted above, many investigators continued to report such findings. These biologically oriented researchers were not part of the internecine battle between homeopathy and traditional medicine. However, there was little attempt to develop a dose response concept consolidation as most of these investigators, a number of whom had become prominent, would migrate into academic administration or government positions leaving the biphasic dose response behind. Thus, there was little effective leadership on this topic. Given the powerful opposition from traditional medicine and its impact on pharmacology and toxicology, the biphasic dose response failed to thrive as a concept during the middle decades of the 20 century 15.

Amidst the various scattered, unfocused and yet surprisingly numerous publications of biphasic dose response relationships emerged the work of Thomas Luckey, a biochemist working with gnotobiotic experimental systems. In the 1950s Luckey 20,21 reported that hormetic dose responses occurred with various antibiotic treatments in poultry. Luckey would eventually turn these and other observations into a general biological concept, not unlike that proposed by Schulz some 70 years earlier, reactivating the dose response debate. However, Luckey’s synthesis would occur with ionizing radiation hormesis rather than chemical hormesis. Substantial examples of radiation and chemical radiation had been reported in the early literature on hormesis. Yet, researchers in these two domains rarely communicated nor cited each other. Nearly 25 years after his initial observation, Luckey 22 would provide the first detailed book on hormesis, with an exclusive focus on radiation. This book would stimulate the electric power industries in Japan and the US to organize the first conference on radiation hormesis in August, 1985.

Parallel to the efforts of Luckey during the 1970s and 1980s were scientists in a variety of disciplines who reported on the occurrence of biphasic dose responses, sometimes using the term hormesis but often with terms such as J- or U-shaped, bimodal, bidirectional, or others. Amongst the leaders was Tony Stebbing, an ecotoxicologist from the UK, who developed a cybernetic regulatory mechanism to account for hormetic dose responses 23. On the pharmacological side, the work of Szabadi 24 was equally important as biphasic dose responses were reported in numerous receptor systems, with the responses mediated via the interaction of different receptor subtypes via the same agonist. Thus by the 1980s the hormesis concept experienced a multidisciplinary resurgence within small pockets of the biological and biomedical sciences.

While there was the renewal of interest in hormesis within the scientific community, interest also emerged from the regulated chemical and electric industry communities. These groups had recently become targeted for regulation using the conservative linear-no-threshold (LNT) dose response model for carcinogens. While the industry initially challenged the legitimacy of the LNT model it was unsuccessful because its alternative to the LNT, that is, the threshold model, was difficult to differentiate from the LNT model when only a few doses were used in hazard assessment cancer bioassay studies. It became clear that there may be a better chance to challenge the LNT model via the use of a hormetic model since the two types of dose responses were more fundamentally different and gave a greater possibility of being differentiated within experimental systems.

This situation was both good and bad for the hormesis concept. It generated interest from the regulated community but also suspicion and opposition from many in the regulatory agencies, especially in the EPA and public health communities. However, regardless of how regulatory agencies might consider hormesis it is a biological concept and could be studied and assessed.

## HORMESIS: A RESURGENCE

The 1990s saw continued interest in the hormesis concept and in the development of more systematic and rigorous assessment of the historical and contemporary dose response literature. Developments of multiple large data bases of hormetic dose responses using rigorous a priori entry and evaluative criteria revealed that hormesis is very general, being independent of biological model, endpoints measured and chemical class. These assessments revealed that hormesis dose responses could occur following either a direct stimulation or via an overcompensation stimulation following an initial disruption in homeostasis. Analysis of more than ten thousand hormetic dose responses indicated that the magnitude of the hormetic dose response was invariably modest, with the vast majority of hormetic dose responses having a magnitude of response less than two-fold greater than the control group 25. Most maximum responses were only about 30-60% greater than the control group. The modest nature of the hormetic dose response is its most distinct feature but also making it more difficult to prove and replicate. Of significance in this regard is that whether the hormetic response occurred via a direct stimulation or via an overcompensation response the quantitative features of the dose responses were similar. This also revealed that it was necessary to study hormesis within a dose-time-response context. Consistent with these observations is a recent report detailing specific mechanisms for 400 hormesis dose responses via various receptors and cell signaling pathways 26. These findings indicate that hormetic dose responses can occur via a broad range of mechanisms and that the quantitative features of the hormetic dose response is also independent of mechanism.

The fact that hormetic dose response features were independent of biological model, endpoint, inducing agent and mechanism was a striking and unexpected cumulative observation based on an enormous amount of data. Given the extensive diversity in biological systems how could one account for such a consistent and integrative series of observations? These findings lead to the conclusion that the hormetic stimulation provided the first quantitative description of biological plasticity. It revealed how much "gain" there is in biological systems. This capacity is modest, only about 30-60%. This trait is one that has been highly conserved as it exists from plants to humans and at the multiple levels of biological organization. Furthermore, we found that hormetic dose response could in fact be mathematically framed within an allometric relationship, similar to parameters that are functions of body weight or surface area 27. Thus, the concept of hormesis and its accounting for the quantitative features of plasticity represents a discovery of a fundamental biological concept. It is also important to note that this basic concept escaped detection by the scientific community for many decades, perhaps another unknown victim of the battle between homeopathy and traditional medicine.

Detailed examinations of major biomedical content areas such as neurosciences 28, immunology 29, tumor cell biology 30, wound healing 31, aging/biogerontology 32, plant biology 33, and others revealed that hormetic dose responses were common. Entire areas of the pharmaceutical industry have been built upon the hormetic dose response including anxiolytic drugs, anti-seizure drugs, memory enhancing drugs and other areas 28. The expanding areas of pre-conditioning and post-conditioning are also founded upon the hormetic dose-response 34.

The hormetic dose response, which is a measure of biological performance for integrated endpoints, imposes a limitation on the magnitude of the induced biological response, thereby affecting the potential for a pharmaceutical induced benefit. This concept has implications for pharmaceutical companies for drug discovery and development as well as how to optimize the design of clinical trials.

Further investigations have assessed the frequency of the hormetic dose response in the biomedical and toxicological literature. Using rigorous a priori and evaluative criteria an assessment over 21,000 articles in three toxicological and life sciences journals over a thirty year period determined that the frequency of the hormetic dose response was 37% 35. Follow up studies using additional new very large independent data bases revealed that the hormetic dose response model was far more effective than either the threshold or the linear dose responses model to make accurate predictions in the low dose zone 36.

## HORMESIS: IN PERSPECTIVE

This summary reveals that the biomedical, toxicology and regulatory communities made a fundamental error on the nature of the dose response in the low dose zone during the early decades of the 20th century. This mistake was the result of multiple factors, including the conflict between homeopathy and traditional medicine as well as the fact that assessing hormetic dose responses is difficult, requiring more doses than normal, greater sample sizes, and a time component along with the heightened need to replicate such findings. During most of the 20th century insights into the generalizability or the quantitative features of the hormetic dose response were not developed. Thus, there were many reasons for the failure of the hormetic dose response to thrive and mature. In contrast, the threshold dose response was readily adopted by the medical community and quickly integrated into education, training, regulation and therapeutics. This rapid transition not only reflected the important role that the dose response would play in these areas but also that the scientific and regulatory communities failed to vet the capacity of the threshold model to make accurate predictions in the low dose zone prior to accepting this model as the default standard. Thus, the scientific and regulatory communities gave a free pass to the threshold model, permitting public health standards to be based upon an unvetted model. Of course, some 60 years later the threshold and its companion linear (LNT) model failed extensive validation tests while only the marginalized and historically excluded hormetic model passed.

The hormetic dose response model is still facing challenges for recognition and acceptance. The challenges start with the fact that all regulations have been built upon on a hazard assessment scheme that assumes the existence of a threshold dose response. Only three high doses are typically used in such testing, making it virtually impossible to observe a hormetic dose response. Thus, the rules of the testing game significantly affect the outcome. It is important to recognize that past testing and regulations were based on incorrect dose response assumptions. The incorporation of hormetic concepts into the hazard assessment process also requires more doses, animals and a heightened need for replication, all significant disincentives. Despite these challenges and obstacles there is a dose response revolution and at the center of it is the hormesis dose response. This is reflected in the rapid and extensive growth of its citations in the biomedical community (i.e. from about 10 citations/year in the 1980s to nearly 6000 in 2013 in the Web of Science/Knowledge) and in its incorporation into leading textbooks in toxicology and pharmacology, something absent during the 20th century. The hormesis concept helps researchers better address the issue of low dose responses, including areas such as enhancing adaptive capacities, slowing down the onset of chronic degenerative diseases and improving biological performance in many other ways. Hormesis was selected many millions of years ago and incorporated into the diversity of life. The biomedical community has now started to exploit it for public health, therapeutic and commercial benefit.
